# Surgical Clipping of Previously Coiled Recurrent Intracranial Aneurysms: A Single-Center Experience

**DOI:** 10.3389/fneur.2021.680375

**Published:** 2021-09-21

**Authors:** Yongtao Zheng, Lili Zheng, Yuhao Sun, Dong Lin, Baofeng Wang, Qingfang Sun, Liuguan Bian

**Affiliations:** Department of Neurosurgery, Ruijin Hospital, School of Medicine, Shanghai Jiao Tong University, Shanghai, China

**Keywords:** coiled recurrent intracranial aneurysms, surgical clipping, recurrence mechanism, coil removal, by pass

## Abstract

**Objective:** This study reviews our experiences in surgical clipping of previously coiled aneurysms, emphasizing on recurrence mechanism of intracranial aneurysms (IAs) and surgical techniques for different types of recurrent IAs.

**Method:** We performed a retrospective study on 12 patients who underwent surgical clipping of aneurysms following endovascular treatment between January 2010 and October 2020. The indications for surgery, surgical techniques, and clinical outcomes were analyzed.

**Result:** Twelve patients with previously coiled IAs were treated with clipping in this study, including nine females and three males. The reasons for the patients having clipping were as follows: early surgery (treatment failure in two patients, postoperative early rebleeding in one patient, and intraprocedural aneurysm rupture during embolization in one patient) and late surgery (aneurysm recurrence in five patients, SAH in one, mass effect in one, and aneurysm regrowth in one). All aneurysms were clipped directly, and coil removal was performed in four patients. One patient died (surgical mortality, 8.3%), 1 patient (8.3%) experienced permanent neurological morbidity, and the remaining 10 patients (83.4%) had good outcomes. Based on our clinical data and previous studies, we classified the recurrence mechanism of IAs into coil compaction, regrowth, coil migration, and coil loosening. Then, we elaborated the specific surgical planning and timing of surgery depending on the recurrence type of IAs.

**Conclusion:** Surgical clipping can be a safe and effective treatment strategy for the management of recurrent coiled IAs, with acceptable morbidity and mortality in properly selected cases. Our classification of recurrent coiled aneurysms into four types helps to assess the optimal surgical approach and the associated risks in managing them.

## Introduction

After the introduction of coil embolization for intracranial aneurysms (IAs) in the 1990s, the number of IAs treated with endovascular treatment has increased significantly because of its low morbidity and minimal invasiveness. Although short-term safety of endovascular treatment is well-established, the long-term recurrence rate of IAs treated by coiling can be as high as 15–34% (1–3). In contrast, the recurrence rate among clipped patients was about 1–3% (4–6). Many reasons contributing to coiled aneurysm recurrence include initial incomplete aneurysm occlusion, regrowth, and coil compaction and migration, which required retreatment to prevent IA rupture or mass effect. The management strategy in these cases differs from treatment of previously untreated IAs and is more challenging. Individualized endovascular treatments of recurrent IAs involve recoiling, stenting, stent-assisted coiling, and the use of flow-diversion devices (1–3, 6, 7). However, many studies demonstrated that surgical clipping is another effective treatment strategy in managing recurrent coiled IAs (8–10). Obviously, clipping of coiled aneurysms requires complex surgical management in case of removing coils or intraluminal thrombus to securely place the clip at the neck of the aneurysms.

Although many studies reported their experience of surgical clipping these aneurysms, few studies specifically describe the morphological changes of recurrent coiled IAs and their corresponding treatment strategies. The present study describes our surgical experience with recurrent, previously coiled IAs over 10 years at a single center. Furthermore, we conducted a literature review of the previous reports to individualize surgical techniques to effectively manage specific intraoperative obstacles according to the morphological characteristics of the coiled IAs.

## Materials and Methods

### Study Design

From January 2010 and October 2020, 1,650 patients were treated for IAs at the department of neurosurgery at our hospital. Of these, 1,320 patients received endovascular coiling as the first-line treatment. Early surgical clipping was performed in those aneurysms with early hemorrhagic complication or failed embolization. After endovascular treatment, assessment of aneurysm occlusion was performed using a three-point Raymond scale: class I, complete aneurysm occlusion without contrast filling; class II, neck remnant; and class III, residual aneurysm sac. Patients were scheduled for surveillance angiographies to evaluate the stability of coiled aneurysms at ~3, 6, 12, and 24 months. Angiographic recurrence was considered if a previously totally occluded aneurysm had a partial recurrence of the neck and/or sac. In addition, an aneurysm was considered to have remnant growth if a subtotal occluded aneurysm was found to have an increased neck remnant or residual aneurysm. Specifically, patients with bleeding during the follow-up period were performed with retreatment immediately. The decision to retreat a recurrent IAs was made by a multidisciplinary team including vascular neurosurgeons and interventional neuroradiologists. Detailed information of demographic characteristics, hospital information, treatment strategies, discharge status, and long-term clinical outcome was recorded.

### Surgical Technique

Before surgery, each patient's medical status was analyzed, including age, WFNS grade, and medical history. The location, size, and morphology parameters of the aneurysms were also considered, as well as the aneurysms' relationship to their parent artery, collateral circulation compensative capacity, and the condition of vasospasm. The ipsilateral pterional approach was available in patients with IAs located in the anterior communicating artery, posterior communicating artery, and ophthalmic segment of the internal carotid artery (ICA). At the same time, ipsilateral proximal ICA was prepared at the neck for proximal control for clipping ophthalmic aneurysm. Ipsilateral lateral suboccipital craniotomy was performed in those patients with vertebral artery–posterior inferior cerebellar artery aneurysms. A directing clipping was performed in aneurysms with enough neck space for clip placement. In cases of sliding or narrowing of the parent artery, clipping was performed after the removal of coils.

### Follow-Up Evaluation and Outcomes

All intraoperative or postoperative complications were reviewed. The modified Rankin scale (MRS) was used to grade outcomes at discharge and follow-up: scores of 3–6 represent unfavorable outcome, and scores of 0–2 mean good outcome. The mean duration of follow-up was 4.1 years (range, 4 months−9 years).

### Statistical Analysis

Continuous variables were represented by mean ± SD, while grouping variables were represented by quantity/percentage. For single-factor analysis, *t*-tests were used for continuous variables while chi-square was applied for grouping variables. All data were analyzed with SPSS version 19.0, and *p* < 0.05 was deemed statistically significant.

## Result

### Characteristic of Patients and Aneurysms

The patient group consisted of nine women and three men with an age range from 31 to 64 years (mean age, 51.3 years). Ten aneurysms were located in the anterior circulation, and two were located in the posterior circulation. The most common aneurysm locations were anterior communicating artery and posterior communicating artery. Six patients were initially treated by endovascular treatment (EVT) because of aneurysm rupture with consecutive subarachnoid hemorrhage (SAH) (Hunt and Hess Grade II in two, Hunt and Hess Grade III in three, Hunt and Hess Grade IV in one); the remainder harbored incidental aneurysms treated prophylactically. The average initial diameter of those IAs was 8.6 ± 4.2 mm in our study.

### EVT and Indication for Surgery

Primary EVT in all patients was performed in our hospital. Eleven aneurysms were treated with coiling only; one aneurysm with stent-assisted coiling. Post-embolization angiograms demonstrated complete obliteration and incomplete obliteration in 10 and 2 aneurysms, respectively. The early surgeries were operated on because of treatment failure in two patients, postoperative early rebleeding in one patient, and intraprocedural aneurysm rupture during embolization in one patient. Specifically, two failed treatment cases are PICA aneurysm and anterior communicating artery aneurysm. After GDC embolization of those two aneurysms, the coil extrusion into the parent vessel resulted in severe stenosis and complete occlusion in PICA and A2, respectively. To prevent the cerebral infarction, clipping with coils moving was performed in those two patients. Late surgical clipping was necessary in six patients with recurrent or regrowth IAs. Furthermore, one patient suffered with SAH and one patient suffered mass effect in the follow-up period, in whom surgical clipping was performed. Overall, surgical clipping only was performed in eight patients and clipping with coils moving in four patients. The mean interval between primary EVT and microsurgical clipping was 13.3 months (range 2–36 months) in those patients.

### Outcomes and Complications

The surgical procedures, results, and outcomes are described in Table 1. Ten patients got a favorable outcome (mRS 0–2), but one was disabled and one died. One death occurred as a result of the procedure-related ischemic cerebral infarction. The disability occurred in a 53-year-old patient who was admitted into the hospital because of SAH resulted from ruptured AcomA aneurysm. This patient was successfully managed with GDC embolization initially but presented with rebleeding 1 month later. She subsequently underwent clipping without coils moving. The patient developed cerebral infarction postoperatively and got an mRS of 4 at discharge. One death occurred in a patient 64 years old with a right ophthalmic artery aneurysm. This aneurysm was discovered during an evaluation for headache. The aneurysm was successfully coiled initially but presented with recurrence seen on routine follow-up angiograms 8 months later. Although aneurysm was clipped successfully, the patient died of severe cerebral infarction. At the end of the procedure, aneurysm occlusion was completed for all cases. At the last follow-up, the outcome was classified as good (mRS ≤ 2) in 10 patients (90.9%) and poor (mRS > 2) in 1 patient (9.1%).

**Table 1:** Patients that underwent surgical treatment of aneurysm after previously coiled embolization.

**Case**	**Age/Sex**	**Aneurysm location**	**Initial presentation**	**Initial size**	**Aneurysm neck width**	**Shape of aneurysm**	**Calcification**	**mRROC**	**Size before surgery**	**Indication for surgery**	**Duration since coiling**
1	31/F	PcomA	Headache	11	3.5	Regular	No	II	2.5	SAH	8 weeks
2	37/F	PICA	SAH	5.5	3.0	Irregular	Partial	/	/	Treatment failure	2 h
3	36/F	PcomA	Headache	15	4.0	Regular	Partial	I	6	Regrowth	4 months
4	58/M	AcomA	Dizziness	6	3.0	Irregular	No	/	/	Treatment failure	1 h
5	61/F	OphA	Blurred Vision	11	4.2	Regular	No	I	/	Mass effect	22 months
6	62/F	BA	SAH	12	5.5	Regular	Partial	I	6	Recurrence	25 months
7	56/F	PcomA	Headache	6	2.5	Regular	No	I	2	Recurrence	7 months
8	53/F	AcomA	SAH	3	1.5	Irregular	No	II	1	Rebleeding	1 month
9	59/M	AcomA	SAH	7	3.0	Regular	No	I	3	Recurrence	3 months
10	64/F	OphthA	Dizziness	16	6.5	Regular	Partial	IIIa	5	Recurrence	7 months
11	46/M	AcomA	SAH	5	2.0	Regular	No	II	/	IPAR	1 h
12	53/F	AcomA	SAH	6	2.5	Regular	Partial	I	4	Recurrence	36 months
**Case**		**Surgical approach**		**Coil situation**		**Radiological outcome**		**Complication**		**mRS**	
1		Clipping		Moving		Complete		/		0	
2		Clipping		Moving		Complete		/		0	
3		Clipping		No Moving		Complete		/		0	
4		Clipping		Moving		Complete		/		0	
5		Clipping		Moving		Complete		/		2	
6		Clipping		No Moving		Complete		/		1	
7		Clipping		No Moving		Complete		/		0	
8		Clipping		No Moving		Complete		Cerebral infarction		4	
9		Clipping		No Moving		Complete		/		0	
10		Clipping		No Moving		Complete		Cerebral infarction		6	
11		Clipping		No Moving		Complete		/		1	
12		Clipping		No Moving		Complete		/		0	

*F, female; M, male; SAH, subarachnoid hemorrhage; OphA, ophthalmic artery; PcomA, posterior communicating artery; BA, basilar artery; AcomA, anterior communicating artery; PICA, posterior inferior cerebellar artery; IPAR, intraprocedural aneurysm rupture. mRS, modified Rankin scale*.

### Recurrent IAs Were Treated Conservatively or Endovascularly

Follow-up imaging was available in 995 aneurysms of the 1,320 treated aneurysms. One hundred and twenty-four aneurysms (12.5%) developed a recurrence after the initial embolization. Excluding six aneurysms treated with clipping in this study, 83 underwent a second embolization, and 35 underwent conservative treatment. Of the 83 aneurysms with a second embolization, 9 aneurysms developed a slight recurrence again; however, conservative treatment was performed in those aneurysms. Thirty-five aneurysms with slight recurrence were performed with conservative treatment. No aneurysmal SAH was found in those patients.

## Discussion

Although high postoperative recurrence rates occurred in patients treated with endovascular coiling for IAs, the treatment strategy of those patients may vary from the morphology and recurrence degree of aneurysms. In our study, four aneurysms were clipped at the early phase because of rebleeding, embolization failure, or intraprocedural aneurysm rupture during embolization, and eight aneurysms were clipped at the late phase because of rebleeding, mass effect, or recurrence. The high rate (100%) of complete obliteration in 12 aneurysms is consistent with previous reports. Two major complications of ischemic stroke were observed in this series of patients, which resulted in death and permanent neurological deficit, respectively. Among these 26 studies spanning 716 patients with 723 aneurysms in the past 20 years, the mortality rate ranged from 0 to 29.6%, the complete obliteration rate of IAs from 73.7 to 100% (Table 2).

**Table 2:** Case series reports on microsurgical management of coiled intracranial aneurysms in the past 20 years.

**References**	**No. of patients and IAs**	**Sex/age (M/mean)**	**Median latency (months)**	**Surgical indication (** ***n*** **)**						**Treatment strategy (** ***n*** **)**			**Mortality (%)**	**Postsurgical obliteration rate (%)**
				**Recurrence**	**ME**	**CM**	**CP**	**Rebleeding**	**Residual aneurysm**	**Clipping**	**By-pass**	**Other treatment**		
Thornton et al. (9)	11/11	2/49	4.36	2	3	2	1	0	3	11	0	0	29.6	NA
Makoui et al. (4)	1/1	0/46	0.5	1	0	0	0	0	0	1	0	0	0	100.0
Asgari et al. (1)	5/5	2/47.2	2.3	2	0	1	0	0	2	5	0	0	0	100.0
Conrad et al. (11)	7/7	1/50	4.43	3	0	0	1	0	3	4	0	3	14.3	100.0
Zhang et al. (6)	38/40	11/52	6	22	0	1	0	0	15	31	3	6	13.2	NA
Veznedaroglu et al. (12)	18/18	3/49	12.8	18	0	0	0	0	0	15	0	3	0	83.3
Minh et al. (2)	7/7	1/42	NA	1	0	0	0	2	4	7	0	0	14.2	100.0
Raftopoulos et al. (13)	17/17	9/54	17.8	14	0	2	1	0	0	17	0	0	0	100.0
König et al. (14)	10/10	2/46	14	6	0	0	4	0	0	10	0	0	0	NA
Tirakotai et al. (15)	8/8	1/49	12.4	2	3	0	0	1	2	7	1	0	12.5	100.0
Lejeune et al. (16)	21/21	11/45	8.33	21	0	0	0	0	0	19	0	2	10.0	90.5
Klein et al. (17)	13/13	6/43	19.6	10	0	0	0	3	0	13	0	0	7.7	100.0
Waldron et al. (3)	43/43	9/51	28	10	0	0	13	0	20	33	7	3	9	76.7
Chung et al. (18)	29/29	16/48.1	3.93	10	0	0	6	5	8	29	0	0	6.9	NA
Romani et al. (19)	81/82	28/47	12	23	3	4	2	4	46	78	2	2	12	93.9
Nakamura et al. (20)	15/15	8/50.6	19.1	12	0	0	0	0	3	15	0	0	13.3	100.0
Rubino et al. (21)	20/20	8/43.5	NA	7	0	0	0	0	13	20	0	0	5	95.0
Izumo et al. (22)	7/7	1/60.3	28.8	5	0	1	0	0	0	5	1	0	0	85.7
Daou et al. (23)	111/111	29/50.5	23	95	0	0	0	2	14	105	0	6	15	97.3
Wang et al. (24)	19/21	9/51.3	26	18	0	0	0	1	3	18	1	2	9.5	85.7
Toyota et al. (25)	14/14	7/50	12	14	0	0	0	0	0	13	1	0	0	78.6
Shtaya et al. (26)	39/40	19/49	18	38	0	0	0	0	2	40	0	0	5.1	NA
Nisson et al. (27)	53/53	7/51.9	31.3	25	0	0	0	0	28	53	0	0	6	94.3
Wu et al. (28)	48/48	26/46.5	20.2	29	9	0	0	4	6	48	0	0	10.4	100.0
Liu et al. (29)	75/76	34/56	7	33	0	0	0	4	39	68	2	4	14.7	73.7
Raper et al. (5)	6/6	3/53	7.5	5	0	0	0	3	0	6	0	0	0	100.0

*ME, mass effect; CM, coil migration; CP, coil protrusion*.

### Mechanisms and Type of IA Recurrence

**Figure 1:**
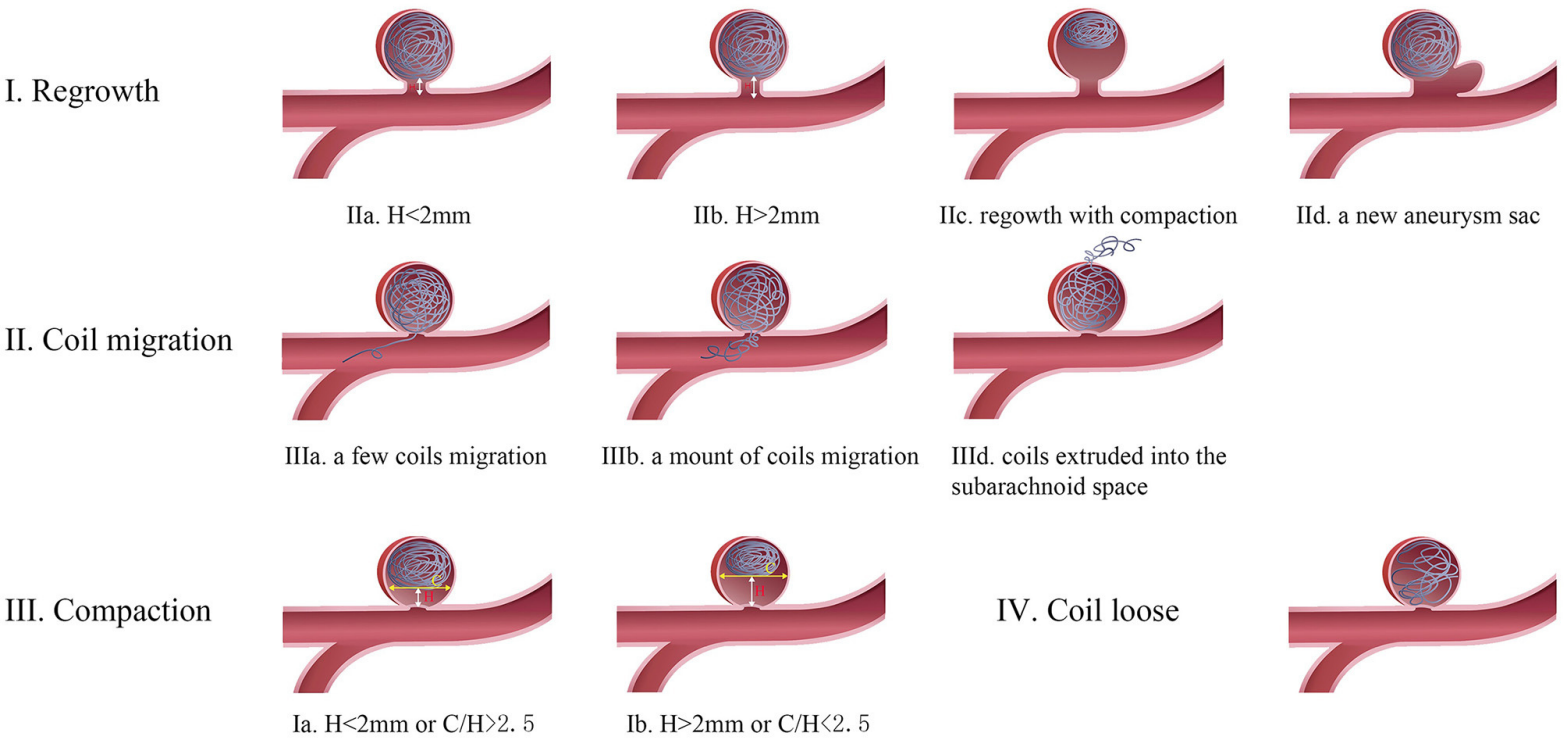
The different types of recurrent aneurysms. Type I. Aneurysm regrowth: neck height <2 mm; neck height more than 2 mm; aneurysm regrowth withe coils compaction; a new aneurysm sac originated from the initial aneurysm neck. Type II. Coil migration: very few coils fill the neck of the aneurysm or the parent artery; multiple coils fill the neck of the aneurysm or the parent artery; coil extrusion into subarachnoid space. Type III. Coil Compaction: compaction height <2 mm or coil width (C) to compaction height (H) ratio > 2.5; compaction height more than 2 mm or C/H < 2.5. Type IV. Irregular recanalization inside the packed coils.

**Figure 2:**
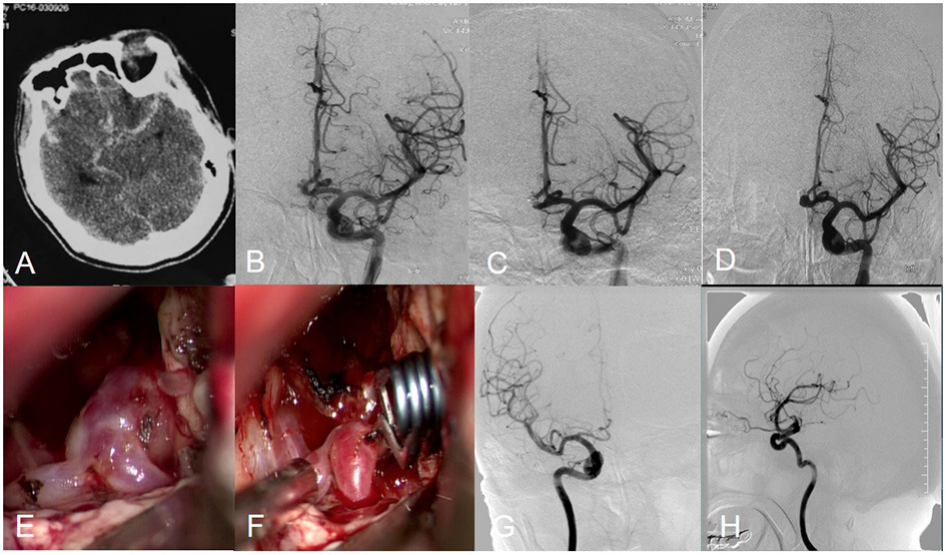
Angiograms obtained in Patient 12, a 53-year-old female with an anterior communicating artery aneurysm. CT scan showed SAH **(A)**. Preembolization angiogram **(B)**. Postoperative arteriogram demonstrating complete obliteration of aneurysm **(C)**. Follow-up angiogram showed aneurysm recurrence **(D)**. Intraoperative photograph showed aneurysm clipping **(E,F)**. Postoperation angiogram showed aneurysm was clipped completely **(G,H)**.

**Figure 3:**
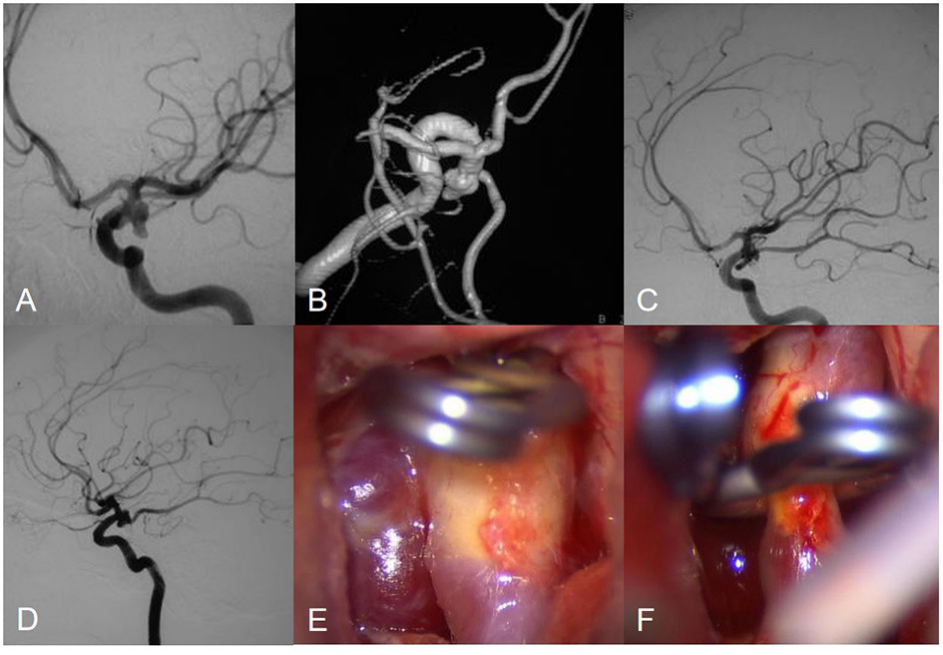
Angiograms obtained in Patient 7, a 56-year-old female with an posterior communicating artery aneurysm. Preembolization lateral angiogram **(A,B)**. Postoperative arteriogram demonstrating complete obliteration of aneurysm **(C)**. Follow-up angiogram showed aneurysm regrowth **(D)**. Intraoperative photograph showed aneurysm clipping **(E,F)**.

**Figure 4:**
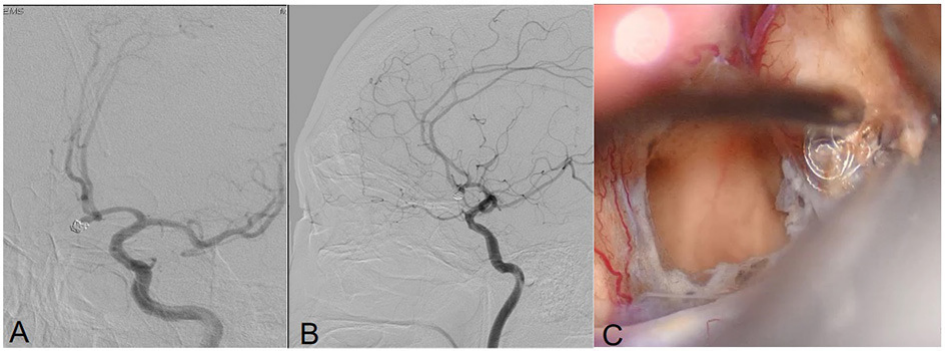
Angiograms obtained in Patient 9, a 59-year-old male with an anterior communicating artery aneurysm. DSA demonstrated recurrent coiled aneurysm **(A,B)**. Intraoperative view of the aneurysm showing the extrusion of the coils **(C)**.

Aneurysm recurrence after endovascular coiling is a common problem, occurring in around of 30% cases depending on the series (12, 13). Many studies reported that the size and neck width of IAs, aneurysm rupture, initially incomplete occlusion, packing densities, the use of stent, and length of follow-up contributed to an increased risk of IA recurrence (4, 5). The mechanisms of aneurysm recurrence after EVT were summarized as follows (Figure 1): coil compaction (Figure 2), aneurysm regrowth (Figure 3), and fundal migration (Figure 4). Coil compaction is reported to be the most important contributing factor to IA recurrence, which was caused by the water hammer effect of the pulsatile flow and dissolution of the thrombus within the IAs sac. The mechanisms of aneurysm regrowth and coil compaction are indistinguishable. Hoppe et al. proposed that aneurysm sac growth was the primary reason for recurrence after successful endovascular coiling; however, there was no association between IA recurrence and coil compaction (30). At the same time, it is difficult to identify coil extrusion from coil compaction in angiography. Four mechanisms of coil extrusion were reported, namely, iatrogenic coil extrusion, initial coiling of a pseudoaneurysm, forcible coil compaction, and degradation of the distal aneurysm wall. Coil extrusion was observed during surgery more frequently than expected, which increased the rupture risk of IAs. In aneurysms with intraluminal thrombus, coils can gradually penetrate into the thrombus, resulting in restoration of flow into the aneurysm lumen.

### Retreatment Indication After Initial Embolization

Despite the high recurrence rate in IAs following EVT, the annual re-rupture rates after endovascular coiling reported in the previous studies range from 0.11 to 0.32% (14–16). Furthermore, Byrne reported a 0.4% rebleeding rate for stable non-progressing aneurysmal remnants and a rebleeding rate of 7.7% for angiographically unstable aneurysmal residuals (7). Therefore, long-term angiography follow-up for detection of recurrences is essential for patients with aneurysms larger than 10 mm and in patients with grade 2 occlusions. Although the previous study showed a very low rupture rate of coiled aneurysms at 10 years or more, data at follow-up of IAs beyond 5 years after endovascular coiling are scant (7–10).

It is critical to balance the morbidity associated with retreatment with rupture risk of the recurrent aneurysm when deciding which patients should undergo retreatment with a coiled aneurysm. The data available thus far demonstrated an equivalent procedural morbidity related with retreatment of coiled IAs with the first treatment, ranging from 0.43 to 3.2% (7–10, 14–16). Of the 127 patients with aneurysm recurrence in a large study reported by Dorfer, 52 patients underwent surgical clipping and 75 underwent re-embolization over an 18-year period. A low rate of treatment-related morbidity and a high technical success rate were found in both surgical and endovascular treatment (8). Therefore, re-treatment of those coiled IAs which are prone to rupture is feasible.

### A Brief Review of Endovascular Retreatment for Coiled IAs

EVT of coiled aneurysms has become a safe and effective option as new coil technologies and assistant devices develop and practitioners subsequently gain experience with these techniques, with the retreatment rate ranging from 6.9 to 17.4% (2, 3). Rebleeding rates after EVT are estimated at between 1 and 2% in larger series (12–14). Endovascular retreatment approaches of aneurysms previously embolized include coiling alone, balloon- and stent-assisted coiling, deployment of covered stents, and flow diversion. IAs with obvious compaction or regrowth and appropriate morphology allow coiling alone as the recurrent cavity is large enough. The stent-assisted technique is suitable for those aneurysms with a shallow recurrent cavity to prevent coils from escaping into the parent artery, or flowing into a distal artery, which results in artery occlusion. Li et al. reported 12 recurrent coiled IAs treated with stent-assisted coiling, no neurologic deficits, or aneurysmal rupture occurring during the follow-up period (31).

Although covered stent was used to treat large or giant wide-necked IAs and carotid-cavernous fistula before, it was also suitable for recurrent aneurysms, regardless of the size and shape of the recurrent aneurysm. The stent's membrane can act as a barricade to prevent the thrombus formed within the aneurysm cavity from entering the blood, which decreases the possibility of thromboembolic complications. Some papers have reported the safety and efficacy of flow diversion for residual or recurrent IAs after EVT (23, 32, 33). In two large retrospective series of recurrent aneurysms treated with flow diversion, the complication rates were 3.0% (1/33) and 10.3% (3/29), respectively (32, 33). Thromboembolism is the most common complication in the endovascular retreatment of IAs, ranging from 0 to 11% in previous series (22, 27). The recurrence rate of IAs after endovascular retreatment is around 10%, which is higher than that of surgical clipping. Although recanalization may occur even after endovascular retreatment, additional re-embolization showed a lower procedure-related complication compared with surgical clipping. Previous series show similar and even lower peri-procedural complication rates than the rates during the initial treatment of IAs. Therefore, it may be reasonable to attempt re-embolization firstly in embolized patients with recanalized aneurysms.

### Surgical Techniques for Coiled IAs

Predicting the clippability of recurrent IAs helps us make surgical planning and determine the timing of surgery, such as clipping the aneurysm immediately or observing a surgery being performed. In this study, we summarize an exhaustive classification scheme for recurrent aneurysms based only on angiographic findings, which might be more systematic and specific for surgical planning. The recurrent IAs can be classified as four groups from a therapeutic perspective: (1) a compaction height (H) of <2 mm or a ratio of coil width to compaction height (C/H) of >2.5; (2) a compaction height of >2 mm or a C/H of <2.5; (3) a few coils across the IA neck; and (4) some coils across the IA neck or extrusion into the parent artery or extrusion outside the IA wall.

**Figure 5:**
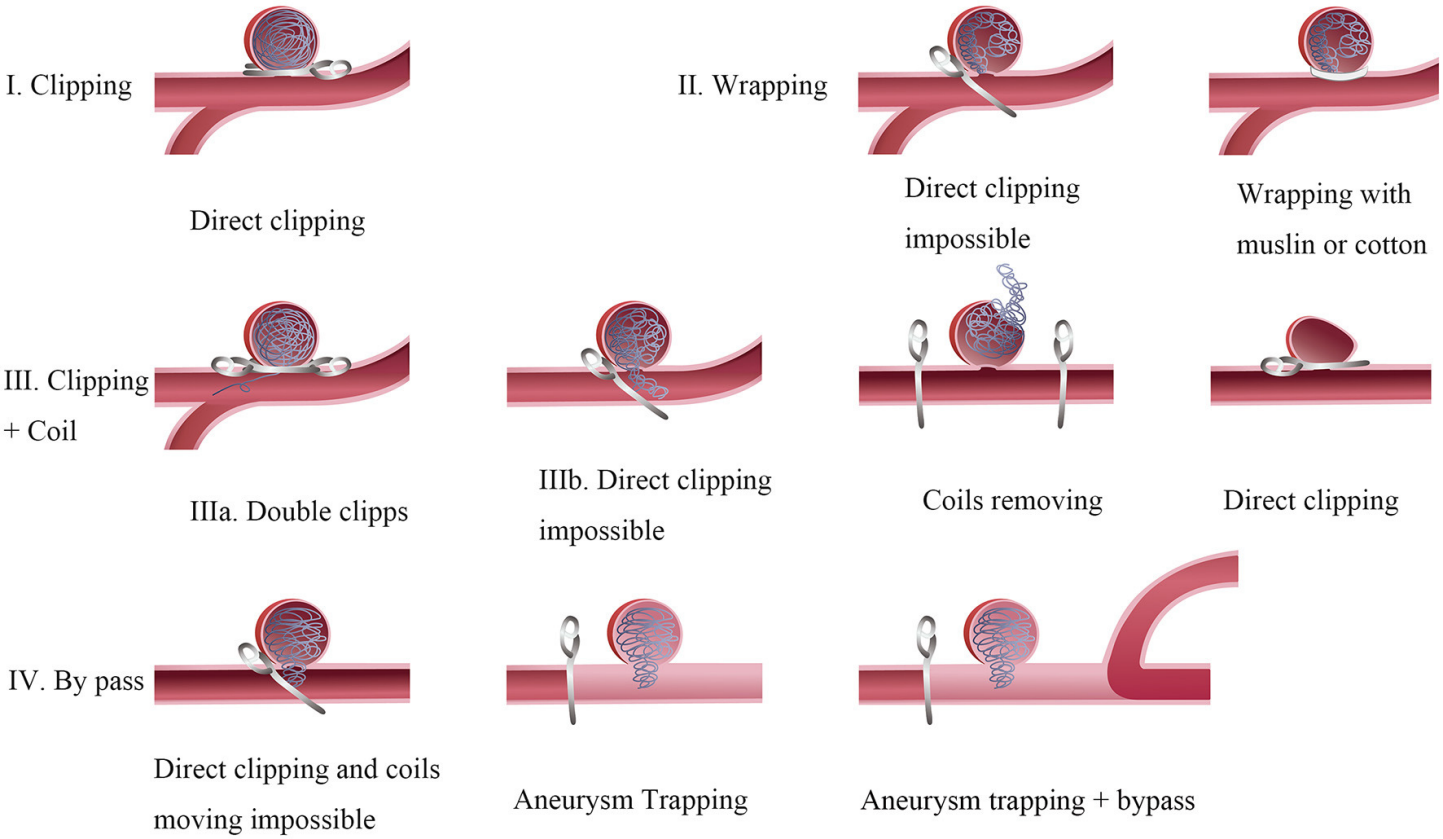
Surgical techniques of recurrent intracranial aneurysms. Type I: Direct clipping is available in those aneurysms with a compaction height >2 mm. Type II: Wrapping aneurysms with muslin or cotton for these difficult cases when multiple clipping attempts failed. Type III: Clipping aneurysms with coils extrusion. Ma: A tandem clipping method helps to the complete obliteration in aneurysms with a few coils extrusion. Illb: Coils moving is inevitable in aneurysms with multiple coils extrusion or mass effect occurring. A temporary occlusion of the parent vessel and expose the aneurysm as much as possible to clarify its relationship with the parent artery. Type IV: Aneurysm Trapping and by pass. Coils moving maybe hazardous in cases after endovascular treatment for a long time. A bypass strategy with parent artery occlusion is suitable for those cases.

For type I aneurysm with minimal compaction or regrowth, conservative observation for further coil compaction or neck remnant regrowth is a preferred choice as there is not enough space for the clip placement (12, 13). Direct clipping requires enough coil-free space at the base of the aneurysm. However, the degree of free base depends on the experience of the surgeon, the morphology characteristics, and even the clip type (14, 16). Direct clipping is available in type 2 aneurysms with a compaction height of >2 mm or a ratio of coil width to compaction height of <2.5, which facilitates safe clip placement, particularly in young patients (22, 27). Wrapping aneurysms with muslin or cotton is another treatment for these difficult cases when multiple clipping attempts failed. Wrapping can induce inflammation and scarring of the aneurysm wall which prevent IAs from enlarging and rupturing (Figure 5) (17, 18, 34).

For type 3 aneurysm, a single clip is hardly to clip the aneurysm completely because of inadequate closure of the far side of the neck by coil extrusion into the parent artery. Tsuyoshi proposed combining a fenestrated clip with another type of clip to complete closure of those complex aneurysms. The former clip is available for closing the far-side aneurysmal neck, while the latter is used to close the near side of the neck (17).

Although removing the coil from the aneurysm sac is always difficult and hazardous, it is inevitable when loops of coil extrusion into the parent vessel make complete clipping impossible in type 4 aneurysms (17, 18, 21, 34). Whether the coils can be successfully removed from the aneurysm or parent artery mainly depends on the time that has elapsed since EVT. An animal experiment demonstrated that a few neoendothelial cells were detected on the implanted coil and neoendothelial cells were more confluent over the coils at 4 and 8 weeks after coil embolization (35). Therefore, attempted coil removal may be very difficult and traumatic if a relatively long time has elapsed after embolization. Opening the aneurysmal sac and removing the coils are possible in the early post-coiling period. If coil removal is necessary during the operation, it is of utmost importance to prepare a temporary occlusion of the parent vessel and expose the aneurysm as much as possible to clarify its relationship with the parent artery (20). It is hard to decide the amount of coil extraction which mainly depends on the doctor's experience to achieve an optimal position of the aneurysm clip. Despite the feasibility of direct aneurysm with removing coils, it has significant disadvantages, such as uncontrollable ischemia time, tearing risk of the arterial wall, and difficulty reconstructing the neck after transecting the aneurysm (24, 25).

In contrast, a bypass strategy with parent artery occlusion can be executed methodically for unclippable aneurysms (19, 24–26, 28). Currently, many types of extracranial-to-intracranial bypasses, including high-flow bypasses with saphenous veins or radial artery grafts, have been developed to revascularize the parent artery of unclippable IAs effectively (29). Coil removal may be inevitable in patients with large or giant aneurysms who present with mass effect symptoms. Thornton et al. proposed that implanted coil combination with subsequent thrombus can also generate mass effects on the parent artery (18). Clipping the coiled giant aneurysms is technically challenging as the dissection and visualization around the lesion are difficult. Therefore, a bypass with aneurysm resection may be preferred other than direct clipping.

A few studies reported about surgical clipping of previously stent-assisted coiling aneurysms, which was associated with higher procedural complication and further technical challenge as the intraluminal stent was embedded in the parent artery wall. Further, temporary clipping is difficult as the vessel becomes more rigid and less maneuverable, with the risk of vessel deformation after withdrawing temporary clipping. In the study by Liu et al., the stent immediately regained its previous shape with no associated compromised flow in three recurrent stent-coiled IAs performed with temporary clipping of the parent artery (29). However, temporary clipping over the stent is likely to be a technical challenge in our opinion unless more data on this issue are available.

### Limitation

There are some limitations to our study. First, this study describes our surgical experience in recurrent, previously coiled IAs over 10 years in our hospital. As the technological levels changed over time, the long time span covered in this study influenced the accuracy of the results to a certain degree. Second, although clinical decision regarding the use of endovascular vs. surgical techniques was made by vascular neurosurgeons and interventional neuroradiologists, there are no standardized procedural protocols and criteria for treatment strategies and timing because of its retrospective design and small sample. Third, the study population was gathered from a single center, which may not always reflect the findings and practices of other hospitals. Lastly, most aneurysms enrolled in our study were relatively easily accessible to clip directly with or without coil removal. More complex aneurysms with high surgical difficulties, such as bypass surgery, trapping, and wrapping, should be admitted in the future.

## Conclusion

Surgical clipping can be a safe and effective treatment strategy for the management of recurrent coiled IAs, with acceptable morbidity and mortality in properly selected cases. In this study, we have presented our experience of dealing with 12 coiled IAs with a relatively low risk and a high rate of complete obliteration. Our classification of recurrent coiled aneurysms into four types (10 subtypes) helps to assess the optimal surgical approach and the associated risks in managing them. Furthermore, we elaborate the different surgical techniques according to the IA recurrence type, including direct clipping, clipping with coils moving, wrapping, and aneurysm trapping with bypass.

## Data Availability Statement

The raw data supporting the conclusions of this article will be made available by the authors, without undue reservation.

## Ethics Statement

Ethical review and approval was not required for the study on human participants in accordance with the local legislation and institutional requirements. The patients/participants provided their written informed consent to participate in this study. Written informed consent was obtained from the individual(s) for the publication of any potentially identifiable images or data included in this article.

## Author Contributions

LB: conception or design of the work. YZ, LZ, and BW: drafting the work. LZ, YS, DL, and QS: acquisition, analysis, or interpretation of data for the work. YZ and LB: agreement to be accountable for all aspects of the work in ensuring that questions related to the accuracy or integrity of any part of the work are appropriately investigated and resolved. All authors: revising it critically for important intellectual content and final approval of the version to be published.

## Funding

This study was supported by the Technology of China and National Natural Science Foundation of China (82001261) and the Shanghai Sailing Program (20YF1403800).

## Conflict of Interest

The authors declare that the research was conducted in the absence of any commercial or financial relationships that could be construed as a potential conflict of interest.

## Publisher's Note

All claims expressed in this article are solely those of the authors and do not necessarily represent those of their affiliated organizations, or those of the publisher, the editors and the reviewers. Any product that may be evaluated in this article, or claim that may be made by its manufacturer, is not guaranteed or endorsed by the publisher.
